# Maternal Anemia during pregnancy and infant low birth weight: A systematic review and Meta-analysis

**Published:** 2017-03

**Authors:** Shoboo Rahmati, Ali Delpishe, Milad Azami, Mohammed Reza Hafezi Ahmadi, Kurosh Sayehmiri

**Affiliations:** 1 *Ilam University of Medical Sciences, Ilam, Iran.*; 2 *Department of Epidemiology, Ilam University of Medical Sciences, Ilam, Iran.*; 3 *Department of Pathobiology, Ilam University of Medical Sciences, Ilam, Iran.*; 4 *Department of Biostatistics, Ilam University of Medical Sciences, Ilam, Iran.*

**Keywords:** Anemia, Hemoglobin, Low birth weight, Pregnancy, Pregnant women, Meta-analysis

## Abstract

**Background:**

Infant low birth weight is one of the major problems in different societies. Different reports have provided different results regarding the relationship between maternal anemia and infant low birth weight in different months of pregnancy.

**Objective:**

The aim of this study was to determine the relationship between maternal anemia during pregnancy and infant low birth weight.

**Materials and Methods:**

This systematic review was conducted using related keywords in national (Sid, Iran.doc, Iran medex and Magiran) and international (PubMed, Science Direct, Cochrane, Medline, Web of Science, Scopus, Springer, Embase, Google scholar) databases. Relative risks and confidence intervals were extracted from each study. The results were combined using random-effects model for meta-analysis. The I^2^ index was also used to measure heterogeneity between the studies.

**Results:**

Overall, 17 studies with a total sample size of 245407 entered the final meta-analysis and demonstrated that the relative risk for maternal anemia in the first, second and third trimester of pregnancy were 1.26 (95% CI: 1.03-1.55), 0.97 (95% CI: 0.57-1.65), and 1.21 (95% CI: 0.84-1.76), respectively. The relationship between maternal anemia and infant low birth weight in the first trimester of pregnancy was significant.

**Conclusion:**

Maternal anemia, especially during the first trimester of pregnancy, can be considered as a risk factor for pregnancy outcomes. Therefore, one needs to take the necessary steps to cure this disease in order to reduce the incidence of infant low birth weight.

## Introduction

Low birth weight (LBW) is a major problem in different societies. On average, 7.7% of infants weigh less than 2500 gr. Infants with birth weight less than 2500 gr are known as LBW infants, regardless of gestational age. According to a study, 10% of infants born in the United States are LBW infants (2). Several maternal factors such as age, anemia, etc. may affect LBW of the infants. Therefore, anemia during pregnancy can be considered as one of the major causes of infant LBW (1, 2). According to the data collected from the Center for Disease Control in 1989, if hemoglobin level in the first and the second trimester of pregnancy is less than 11 g/dL and is less than 10.5 g/dL in the third trimester, this condition is considered anemia (3). According to the recommendations of World Health Organization (WHO) in 1972, a pregnant woman is diagnosed with anemia when the hemoglobin level is below 110 g/l. Based on this report, more than 50% of women who do not take supplements suffer anemia (4). Statistics show that 14-62% of women in developing countries, and 16-29% in developed countries are suffering from anemia (5-7). LBW infants are more vulnerable and exposed to different health problems and complications compared with infants with normal weight. Based on other reports, there is a direct relationship between Low levels of hemoglobin during pregnancy and high birth weight (8, 9). 

Considering the high prevalence of anemia among pregnant women aged 17-45, contradictory results regarding the effect of maternal anemia on birth weight and absence of a definite conclusion in obstetrics and genealogy books, we decided to investigate this topic using meta-analysis methods (10, 11). These methods are able to put together all the facts, thus presenting a more clear and accurate picture of the problem to provide a scientifically proved advice for pregnant women (13, 14).

## Materials and Methods

**Data sources**

This study was a systematic review of the literature and methods based on PRISMA guideline from the beginning of 1990 until April 2017 (15). Valid keywords were used for searching in national (Sid, Iran.doc, Iran medex and Magiran) and international (PubMed, Science Direct, Cochrane, Medline, Web of Science, Scopus, Springer, Embase, Google scholar) databases. In order to maximize the comprehensiveness of the study, general Persian keywords and all possible combinations (maternal anemia and LBW) were used in the search. We used English equivalents such as “anemia”, “LBW” and “pregnancy” in international databases and the “*either/or”* structure was applied for further searching; references were also examined to find further necessary sources on the subject. 

**Study selection (Inclusion and exclusion criteria)**

STROBE, which is a standard and famous international checklist, was used to determine the quality of the study (17). This checklist contains 22 sections, covering various aspects of the study including methodology, sampling, variables measurement, statistical analysis, modification of confounders and the reliability and validity of the tools used to evaluate the study objectives. 15.5 was considered the minimum attainable score. Finally, studies that attained the minimum score of the STROBE checklist entered the study and their data were analyzed using meta-analysis method.

**Study selection**

First, researchers prepared a list of topics and abstracts of each study at the beginning of study and studies that covered the relationship between maternal anemia and LBW based on pregnancy month entered the main study. Cohort, case-control and cross-sectional that evaluated the relationship between maternal anemia and LBW were analyzed in the present study. Relative risk with 95% confidence interval was used to assess the significance of the study.

**Data extraction**

Data extraction was performed by two researchers independently and data extraction form (containing name of the author, year of publication, country, continent, number of participants, trimester and LBW pregnancy outcomes) was used to minimize bias and error in collecting data. In cases of necessity, ambiguity of article information and some certain questions, we contacted the authors by E-mail. The data extracted by the two researchers was compared and discussed in case of possible discrepancies; the dispute was shared with a third person and eventual consensus was reached through re-examination of the issue. 

**Risk of bias assessment**

One of the risks of bias assessment was the fact that data extraction was conducted by two researchers. There was also the risk of dispute.

**Outcomes** **of** **interest**

LBW defined as an infant born at less than 2,500 gr. And hemoglobin level in the first and the second trimester of pregnancy is less than 11 g/dL and is less than 10.5 g/dL in the third trimester, this condition is considered anemia (3).

**Statistical** **analysis**

In present study, the extracted data included study design, type of the study, name of the author, year of publication, continent, number of participants, trimester and the outcome of pregnancy (LBW). The level of hemoglobin was classified in three groups of <14, 11-13, 11> g/dL, while <11 was considered maternal anemia. LBW, as an outcome of pregnancy, was analyzed when the infant weighed less than 2500 grams. The effect of hemoglobin was evaluated using relative risk index (RRI) in three different phases (first, second, and third trimester) of pregnancy based on the weight of the infant. I^2^ index and Q test were used to assess the heterogeneity. Considering the heterogeneity of the studies, random effects model was used to combine different results. STATA Ver 3.2 was used for data analysis and p<0.05 was considered significant for all analyses. 

The minimum and maximum relative risks (RR) or odds ratio (OR) at upper and lower limits of 95% confidence intervals were extracted for each study. In studies, for which this index was not extracted (articles without the OR to use the formula), the index was calculated using OR=ad/bc formula and OR or RR logarithm were used to symmetrize the effect size. Error standard was calculated using the following formula: SE= 1n (upper 95% CI /lower 95% CI) /(2×1.96). If only upper and lower limit had been reported in studies, OR= ad/bc was used. Meta-regression was used to investigate the relationship between the year of study, effect size and causes of heterogeneity. Beggs Funnel Plot was used to examine the publication bias. Sensitivity analysis was used to assess the effect of each study on the overall result.

To analyze sensitivity, the effect of each article was excluded and the result was combined with other studies. This shows whether exclusion of this study has significant effect on the overall result or not. In the case of our study, the effect was not statistically significant.

## Results

**Diagram 1:**
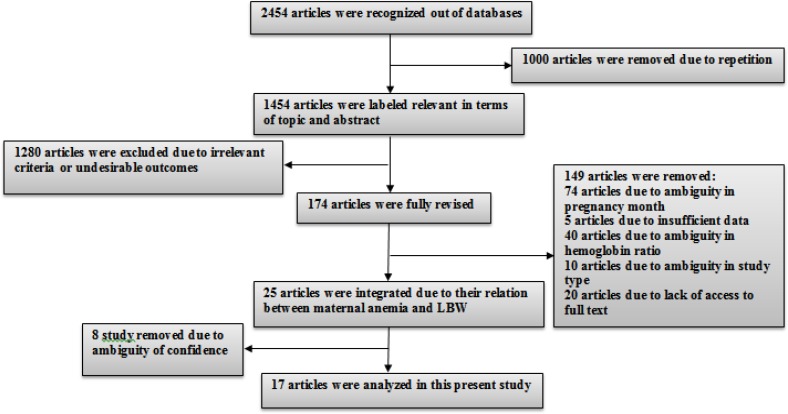
Entry procedures of studies into meta-analysis study

Overall, 2454 relevant articles were identified by searching databases. Studies that did not yield any helpful and relevant information were excluded; 76% of them were cohort studies with an age range of less than 30 years old. 70% of articles were conducted in Asia. (Diagram 1, Table I)

**The relationship between maternal anemia in the first trimester of pregnancy and LBW**

**By type of study**

**Figure. 1:**
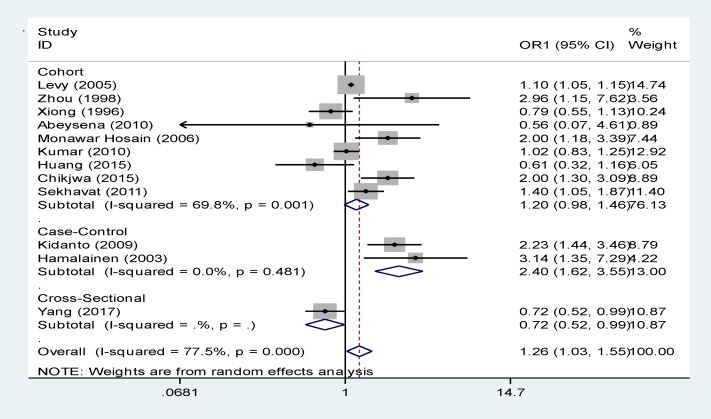
The relationship between maternal anemia and LBW in the first trimester based on the type of study

As illustrated in Figure 1 and table II, there were 9 cohort studies in which the relationship between maternal anemia in the first trimester and LBW was investigated. After combining cohort studies using random effects model, a no significant relationship was observed between maternal anemia in the first trimester of pregnancy and LBW (relative risk, 1.20 [95% CI: 0.98 to 1.46]) (8, 18, 19, 22, 23, 24, 28,29,33). It seems that Levy’s study has estimated this relationship with a relatively high RR (8). There were 2 case-control studies about the relationship between maternal anemia and LBW indicating a significant relationship with a RR of 2.40 (95% Cl: 1.62-3.55) (11, 12). 

It seems that Hamalainen’s study has estimated this relationship with relatively high risk (12). And There were 1 cross-sectional studies about the relationship between maternal anemia and LBW indicating a no significant relationship with a RR of 0.72 (95% Cl: 0.52-0.99) (30). However, the total combination of cohort, case-control and cross-sectional studies using random effects model showed a significant relationship between maternal anemia and LBW in the first trimester with a RR of 1.26 (95% Cl: 1.03-1.55) (8, 10, 11, 18, 19, 22-24, 28-30, 33).

As illustrated in Figure 1, I^2^ index showed that the results of 9 cohort studies were heterogeneous and this heterogeneity was significant (I^2^ Index=69.8%, p=0.001). The heterogeneity of 2 case-control studies was 0.0% and this heterogeneity was not significant (p=0.481). Ov each article was measured individually and it was found that their weight is calculated when the data is combined. Among cohort studies, Levy’s study had the highest weight because of large sample size (8). Kidnato’s study had the highest weight among case-control studies (11).

**By type of** **continent**

**Figure 2:**
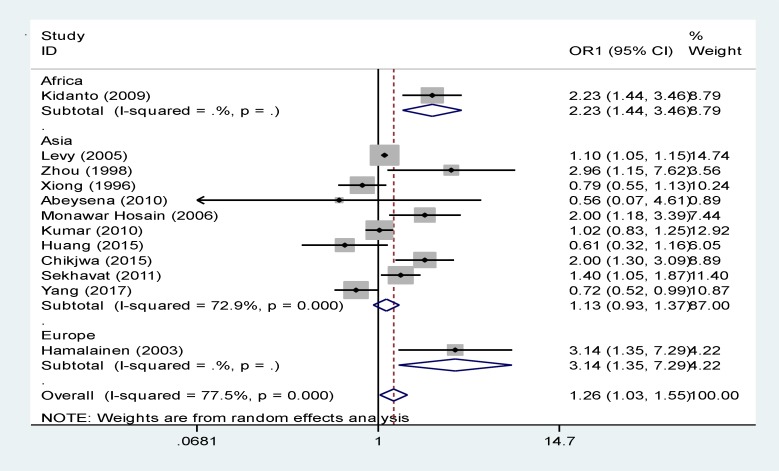
The relationship between maternal anemia and LBW in the first trimester based on continent

Table II and Figure 2 shows the data analysis based on the continent; about 10 studies were conducted in Asia, 1 in Europe and 1 in Africa. RR, confidence interval, and P value were measured through random effects model, that combining Asian studies using a random effects model and this relationship was no significant (relative risk, 1.13 [95% CI: 0.93 to 1.37]). The data related to European studies (relative risk, 3.14[95% CI: 1.35 to 7.29]) and African studies 2.23 [95% CI: 1.44 to 3.46]) are shown in Table II and Figure 2 separately. Levy’s study in Asia had the highest weight (8). 

**By year of publication**

**Figure 3:**
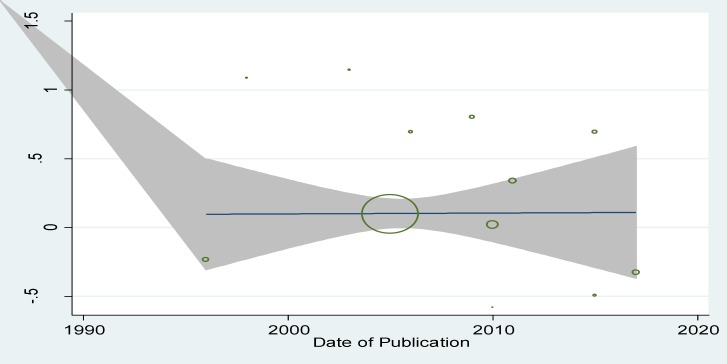
The relationship between maternal anemia and LBW in the first trimester based on the year of publication

According to Figure 3 and Table III, there is not significant relationship between publication year and logarithm size and the the regression equation was estimated to be y=41.52-0.02x. Since p-value was found to be 0.43 for the slope of the line.

**By Begg’s funnel plot**

**Figure 4:**
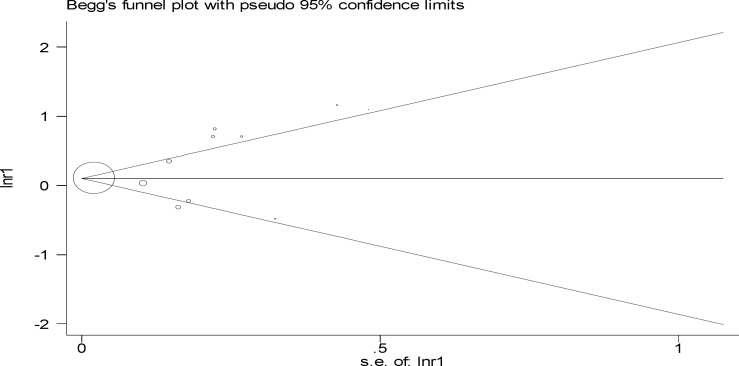
Begg’s funnel plot for relationship of maternal anemia with low birth weight in the first trimester of pregnancy

Figure 4 was used to find the direction of publication bias, according to which the publication direction effect was significant (p=0.02). 

**By Sensitivity analysis**

**Figure 5:**
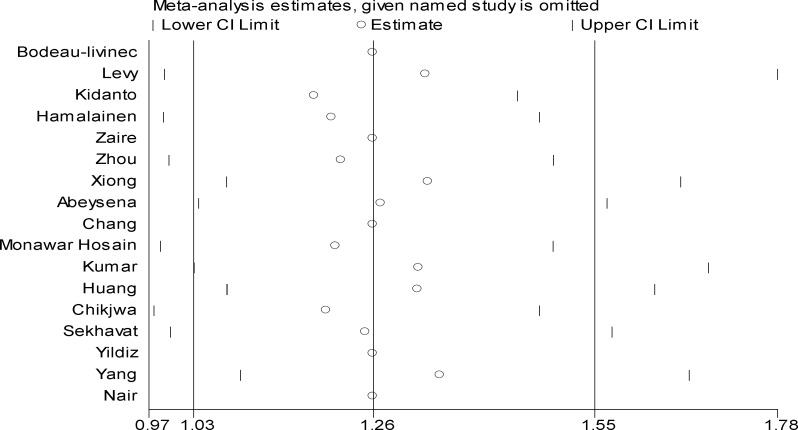
Sensitivity analysis for relationship between maternal anemia with low birth weight in the first trimester of pregnancy

Sensitivity analysis showed that the elimination of a study can either change the results or does not affect the final direction at all and it shows the influence of studies in meta-analysis research. Sometimes, excluding a study can change the overall effect of meta-analysis significantly. In this study, as shown in the figure 5, excluding a study had no effect on the overall results.

**The relationship between maternal anemia and LBW in the second trimester at birth**

**Figure 6:**
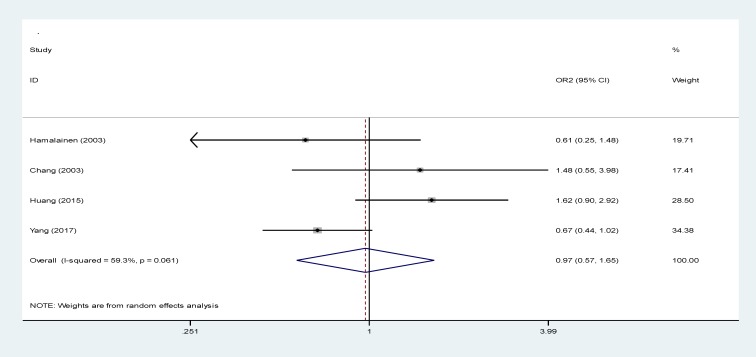
The relationship between maternal anemia and LBW in the secondary trimester of pregnancy

According figure 6 data analysis, 4 studies investigated this relationship in the meta-analysis with a RR of 0.97 (95% CI: 0.57-1.66), while this relationship was not significant. I^2^ index for both studies was measured to be 59.3%, showing a homogenous relationship between the 4 studies. The homogeneity was significant (p=0.0061) (10, 20, 29, 30).

**Relationship between maternal anemia and LBW in the third trimester**

**Figure 7:**
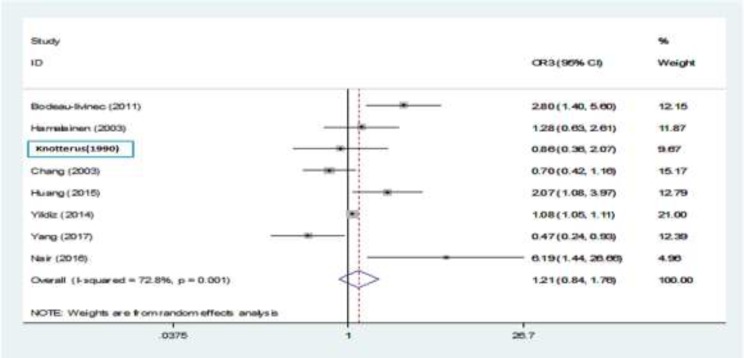
The relationship between maternal anemia and LBW in the third trimester of pregnancy

Of 8 studies that discussed this issue, only 4 studies showed a significant relationship (9, 29, 31, 32). Then, we combined studies using a random effects model and no significant relationship was observed between maternal anemia in the third trimester of pregnancy and LBW (relative risk, 1.21 [95% CI: 0.84 to 1.76] (9, 10, 16, 20, 29-32). Yildiz’s study had the highest weight (31). I^2^ index between this studies was 72.8%, which shows a significant heterogeneous relationship between them (p=0.001) (Figure 7).

**Table I:** Characteristics of studies that entered meta-analysis

**Author-publication year-reference**	**Study design**	**Sample size**	**Mean age**	**Country**	**Trimester**	**RR (lbw)**	**CI-**	**Confounding factors**
Levy-2005 (8)	Cohort	153396	28.3±5.9	Israel	First	1.10	1.10-1.20	Ethnicity, maternal age, placental problems, caesarean delivery, and no vertex presentation
Bodeau-livinec-2011 (9)	Clinical trail	1508	<30	Benin	Third	2/8	1.40-5.6	-
Hamalain-2003 (10)	Case-control	22799	28.9±5.9	Finland	First Secondary Third	3.14 0.61 1.28	1.35-7.28 0.25-1.48 0.63-2.62	No specify
Kidnato-2009 (11)	Case-control	1721	24	Tanzania	First	2.23	1.45-3.5	No specify
Knotterus -1990(16)	Cohort	796	<30	Netherland	Third	0.86	0.35-2.03	-
Zhou-1998 (18)	Cohort	829	25.5±3.8	China	First	2.96	1.15-7.62	No specify
Abeysena-2010 (19)	Cohort	817	26.4±5.5	Srilanka	First	0.56	0.07-4.74	No specify
Chang-2003 (20)	Cohort	918	16.1±1.1	USA	Secondary Third	1.48 0.70	0.55-3.98 0.42-1.25	Parity, BMI, smoking, preeclampsia, and antenatal care
Monawar hosian-2006 (22)	Cohort	350	NA	Bangladesh	First	2	1.18-3.40	No specify
Xiong-1996 (23)	Cohort	16936	25±2.8	China	First	0.79	0.55-1.12	Hospital stay, maternal age, maternal education, parity, gestational age at the first prenatal visit, BMI, hypertensive disorder in pregnancy, vaginal bleeding, and prior spontaneous abortion
Kumar-2010 (24)	Cohort	2027	24.6±3.82	India	First	1.02	0.83-1.25	Maternal age, parity, maternal height, maternal weight, BMI, and gestational age
Sekhavat-2011 (28)	Cohort	1842	-	Iran	First	1.40	1.15-2.06	-
Huang -2015 (29)	Cohort	500	-	China	First Secondary Third	0.61 1.62 2.07	0.32-1.15 0.89-2.90 1.08-3.97	-
Yang-2017 (30)	Cross-sectional	7375	-	china	First Secondary Third	0.72 0.67 0.47	0.50-0.95 0.42-0.98 0.24-0.93	-
Yildiz- 2014 (31)	Cohort	28600	-	turkey	Third	1.08	1.05-1.11	-
Nair - 2016 (32)	Cohort	1007	-	India	Third	6.19	1.44-26.71	-
Chikjwa-2015 (33)	Cohort	1986	-	China	First	2	1.30-3.10	-

Median

LBW: low birth weight

RR: Relative risk

CI: Confidence interval

**Table II:** The relationship between maternal anemia and LBW in the first trimester based on the continent and study type

**Variable**		**Number of studies**	** OR**	**95%CI**	**I** ^2^ ** %**	**P (heterogeneity)**
Type of study						
	Cohort	9	1 .20	0.98-1.46	69.8	0.001
	Case-control	2	2.40	1.62-3.55	0.0	0.481
	Cross-sectional	1	0.72	0.52-0.99	-	-
	Mixture of studies	12	1.26	1.03-1.55	77.5	0.000
Continent						
	Asia	10	1 .13	0.93-1.37	72.9	0.000
	Africa	1	2.23	1.44-3.46	0	-
	Europe	1	3.14	1.35-7.29.	0	-
	Aggregate of continents	12	1.26	1.03-1.55	77.5	0.000

OR: odds ratio

CI: confidence interval

P: p-value

**Table III:** The relationship between maternal anemia and LBW in the first trimester by continent based on year of publication and sample size

		**Coefficients**	**STD. Err** *	**P-value**
Model 1	Date of Publication	-0.02	0.025	**0.43**
	Constant	41.52	50.3	**0.42**

* standard error of coefficients. P-value computed using T statistics

## Discussion

The present study is the first systematic review and meta-analysis research about the relationship between maternal anemia and LBW. Different studies with different values of relative risk and confidence interval were previously conducted (8-11, 16, 18-20, 22-24, 28-33). Various maternal criteria such as anemia, age, intervals between pregnancies, physical tumor factor and weight loss were included in this study. The present study investigated the relationship between maternal anemia and infant LBW.

According to the results, the relationship between maternal anemia and LBW in the first trimester of the pregnancy is significant. maternal anemia causes LBW in the first trimester of the pregnancy. However, this relationship was not significant for the second and third trimesters of the pregnancy. Nowadays, maternal anemia is considered as a public health problem in the world, especially in developing countries. For example, Statistics show that 14-62% of women in developing countries, and 16-29% in developed countries are suffering from anemia (5-7). That the analysis also showed that this relationship is commonly observed in Asia; i.e. LBW is widely seen in Asian countries compared to developed countries according to a study by Lee *et al* (21, 25). Sukrat and Haider’s review article, which analyzed the relationship between density of hemoglobin and outcomes of pregnancy in 2013, showed that hemoglobin less than 11 g/dl increases LBW risk in the first trimester, which is consistent with the present study (26, 27). The present results of meta-regression analysis for the publication year of study showed that there is no statistically significant relationship between publication year of study with the effect size (p=0.43). 

Publication bias, which was mostly observed in meta-analysis and review studies, was due to the fact that published articles did not represent the all researches carried out in that specific area. Actually, articles with significant results, high quality precise design and extensive sample size had higher chances of publications. Another reason is that both native and English languages are used and allowed in meta-analysis studies. According to the results, bias had played a key role in this type of publications. Usually, studies which are conducted according to the norms of relationship assessment increase the chance of bias because articles with positive results are more likely to get published and they are more widely searched.

**Limitations**

1. Exclusion of the relevant and new studies that were not eligible based on inclusion criteria (in terms of Epidemiology)
2. Inability of databases to search using combined keywords
3. Some studies were excluded due to low quality and insufficient proofs
4. Exclusion of some studies due to ambiguity in the month of pregnancy
5. Different definitions for anemia in different studies
6. Exclusion of some studies and articles due to improper direction of publications and lack of causative relationship.

## Conclusion

Considering the relationship between maternal anemia and LBW in the first trimester of pregnancy, anemia can be used as a preventive factor for infant LBW. Therefore, we need to cure maternal anemia to prevent or cure LBW.
